# Prognostic value of molecular markers and cytogenetic alterations that characterize breast cancer precursor lesions (Review)

**DOI:** 10.3892/ol.2013.1589

**Published:** 2013-09-17

**Authors:** MAURIZIO DI BONITO, MONICA CANTILE, ROSSELLA DE CECIO, GIUSEPPINA LIGUORI, GERARDO BOTTI

**Affiliations:** Pathology Unit, National Cancer Institute, INT Pascale Foundation, Naples I-80131, Italy

**Keywords:** breast cancer precursor lesions, molecular alterations, tumor progression

## Abstract

The understanding of the molecular mechanisms that underlie all stages of tumor progression in breast cancer (BC) represents an important goal in the biomedical research of this disease, particularly for the identification of more specific targeted therapies. In this context, BC preinvasive and precursor lesions represent a major dilemma. These lesions are well characterized under the phenotypic and genotypic profile, but it is not clear if they represent obligatory passages of a multistep process determining breast cancer evolution. In fact, the numerous cytogenetic and molecular alterations identified are not always representative of the progression into invasive phenotypes.

## 1. Introduction

Preinvasive and premalignant lesions of the breast are defined as ‘precursor lesions’ and represent a group of extremely heterogeneous lesions that have yet to be clearly classified. In addition, the risk of the neoplastic evolution of the lesions is not yet completely known. At present, these lesions are defined only as ‘risk indicators’ for breast cancer (BC), i.e. lesions with a variable potential to progress to malignant phenotypes. BC precursor lesions include atypical ductal hyperplasia (ADH), atypical lobular hyperplasia (ALH), columnar cell lesions (CCLs), ductal carcinoma *in situ* (DCIS), flat epithelial atypia (FEA), usual ductal hyperplasia (UDH) and lobular carcinoma *in situ* (LCIS) (1).

**Figure 1:**
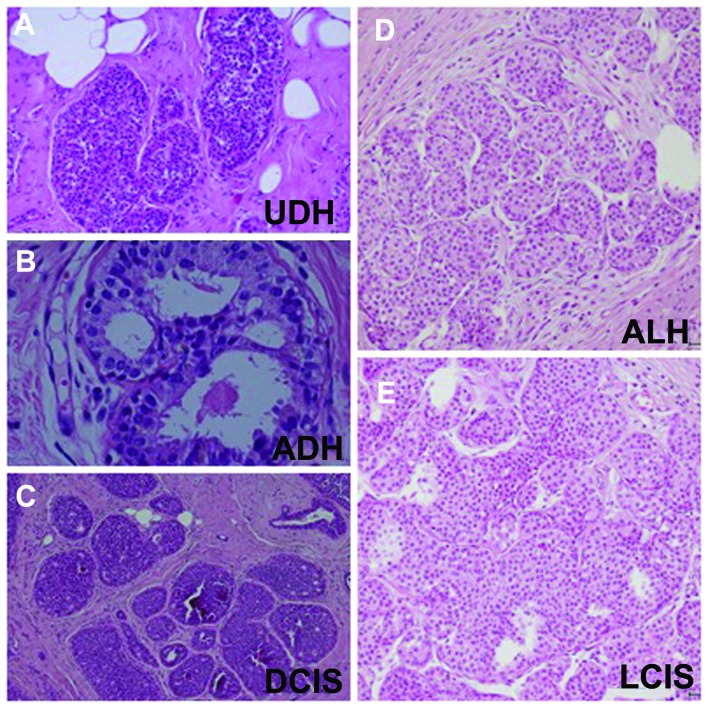
Hematoxylin and eosin (H/E) representation of breast cancer (BC) precursor progression. Ductal precursors: (A) UDH, (B) ADH and (C) DCIS. Lobular precursors: (D) ALH and (E) LCIS (magnification, ×20). UDH, usual ductal hyperplasia; ADH, atypical ductal hyperplasia; DCIS, ductal carcinoma *in situ*; ALH, atypical lobular hyperplasia; LCIS, lobular carcinoma *in situ.*

The molecular and morphological features of these lesions are indicative of a multistep model of breast tumorigenesis that provides a transition from normal epithelium to invasive carcinoma (1). Sequential steps differ according to whether the progenitor cells originate from the ducts or lobules: Ducts, UDH vs. FEA vs. ADH vs. DCIS vs. IDC (invasive ductal carcinoma); and lobules, HL vs. ALH vs. LCIS vs. ILC (invasive lobular carcinoma; Fig. 1) (1).

Consistent with the ‘clonal evolution’ hypothesis, there are numerous studies that show common genetic alterations among the precursors of low-grade BC (low-grade DCIS and ADH) (2).

However, it has also been shown that these lesions do not have the same probability for progression into invasive carcinoma (3).

Recently, as a result of the use of specific and sophisticated technologies, a wide range of molecular alterations associated with preneoplastic forms have been identified. Although they represent a ‘risk of evolution’, their real diagnostic and prognostic value remains unclear.

## 2. Cytogenetic and molecular alterations in precursor lesions

Genotypic-phenotypic potential correlations are consistent with the hypothesis of a multistep evolution of precursor lesions. Several allelic imbalances have been identified in CCLs, located at 3p, 9p, 10q, 11q, 16q, 17p and 17q (4). Moreover, a number of copy number alterations have been described, including the loss of 16q and chromosome X and the gain of 15q, 16p, 17p and 19q (5). ALH and LCIS have a number of common cytogenetic abnormalities, in particular, the loss of 16q, 16p and 17p and the gain of 6q (6).

In addition, ADH and DCIS exhibit extremely similar cytogenetic patterns consistent with the hypothesis of progression. These cytogenetic abnormalities largely involve the loss of 16q and 17p and the gain of 1q (7). With regard to alterations in the expression of specific molecular markers, in certain cases localized in areas that exhibit the chromosomal aberrations described, the estrogen receptor (ER) must be considered. Not all precursor lesions show a positivity for ER, but in the majority of cases, its presence is an important risk factor, particularly if associated to the Ki67 expression, as in DCIS (8). By contrast, ERβ expression is decreased during the supposed precursor lesion progression steps (9). In addition, progesterone receptor expression represents a marker of progression, particularly if associated with ER positivity. Furthermore, its expression appears to correlate with histological grade, but not to the prognosis of IDC patients (10).

With regard to HER-2/c-erb2, hyperexpression/amplification has been detected in ADH and in 25% of LCIS cases if associated with invasive components (11). In addition, the aberrant expression of various molecules of the cell cycle, including p21, p27 and p16, has been identified in BC precursor lesions (12). An additional molecular marker associated with BC progression is Bcl-2. In particular, its expression decreases gradually during the steps of BC tumor evolution and its negativity has been associated with poor prognosis (13). Moreover, Bcl-2 positivity has been identified to correlate with p53 negativity in normal breast tissue and several BC precursor lesions (14). Bcl-2 expression is often also associated with Ki67 expression, in fact, high Ki67 expression, associated with negativity for Bcl-2, has been reported to correlate with the development of poorly-differentiated carcinoma. By contrast, alterations in p53, well described in invasive BCs, are present in DCIS only (15).

E-cadherin is a molecular marker that is well characterized in BC precursor lesions, mainly as it is located in the 16q chromosomal region where losses are commonly described (16).

Finally, epigenetic modifications have been reported to be associated with BC precursor lesions, in particular, gene methylation. E-cadherin hypermethylation has been highlighted in DCIS and the methylation pattern of this gene is consistent with the hypothesis that DCIS represents an IDC precursor (17).

## 3. Conclusions

Molecular techniques, including DNA microarrays, immunohistochemistry and cytogenetic analysis, have made a significant contribution, in a number of cases, to the definition of specific molecular alterations that are common to several lesions at a ‘high risk’ of evolution (18).

However, cellular models suitable for functional studies on the molecular alterations identified, are extremely limited and not representative of the numerous preinvasive/precursors lesions of BC. Furthermore, in the majority of cases, the variable molecular signatures of precancerous lesions are not associated with transformation to the invasive phenotype (3). Therefore, the specific and defined molecular steps are not necessarily identified.

Thus, the existence of a multistep process of evolution of BC precursors lesions remains unknown, preventing the assignment of valid prognostic value to the molecular alterations associated.
